# Effects of Supplementation with Royal Poinciana Seed Meal (*Delonix regia*) on Ruminal Fermentation Pattern, Microbial Protein Synthesis, Blood Metabolites and Mitigation of Methane Emissions in Native Thai Beef Cattle

**DOI:** 10.3390/ani9090625

**Published:** 2019-08-29

**Authors:** Anusorn Cherdthong, Rittikeard Prachumchai, Metha Wanapat, Suban Foiklang, Pin Chanjula

**Affiliations:** 1 Tropical Feed Resources Research and Development Center (TROFREC), Department of Animal Science, Faculty of Agriculture, Khon Kaen University, Khon Kaen 40002, Thailand; 2 Faculty of Animal Science and Technology, Maejo University, Chiangmai 50290, Thailand; 3 Department of Animal Science, Faculty of Natural Resources, Prince of Songkla University, Songkhla 90112, Thailand

**Keywords:** efficiency of microbial N synthesis, enteric methane, feed processing, plant phytochemical, protozoa population

## Abstract

**Simple Summary:**

Strategic feeding of ruminants with pellets containing phytochemical compounds is one approach that could enhance rumen fermentation and decrease greenhouse gases. It was found that feeding with the pellets containing royal poinciana seed meal at 150 g/d might be an alternative to improve rumen fermentation efficiency and reduce the environmental impact of ruminants.

**Abstract:**

The object of this present work was to determine the effects of supplementation with pellets containing royal poinciana seed meal (PEREM) on feed use, ruminal fermentation efficiency, microbial protein synthesis, blood metabolites and mitigation of methane (CH_4_) emissions in cattle. The animals used in this experiment were four male Thai native beef cattle (*Bos indicus*) with initial body weights (BWs) of 125 ± 5.0 kg. Each of the animals were randomly assigned to receive PEREM doses at 0, 50, 100 and 150 g/d, respectively, according to a 4 × 4 Latin square design. Concentrates were fed at 0.5% BW daily, and rice straw was fed *ad libitum*. There were no significant differences (*p* > 0.05) on intakes of rice straw, concentrate and total diet. The intake of nutrients did not change among the levels of PEREM supplementation (*p* > 0.05), except for an intake of crude protein, which was linearly enhanced when increasing the dose of PEREM (*p* < 0.05). The inclusion of different doses of PEREM did not adversely affect the digestibility of dry matter, organic matter, crude protein, neutral detergent fiber and acid detergent fiber (*p* > 0.05). Adding various doses of PEREM did not alter ruminal pH and ruminal temperature, while concentrations of ammonia-nitrogen were significantly increased with an increased dose of PEREM supplementation (*p* < 0.01). The increasing doses of PEREM linearly reduced protozoal numbers (*p* < 0.01), with the lowest concentration when PEREM was added at 150 g. PEREM supplementation did not change (*p* > 0.05) the concentration of acetic acid or butyric acid or the ratio of acetic acid to propionic acid. Nevertheless, the total volatile fatty acid and propionic acid content were changed among PEREM levels (*p* < 0.05), which were linearly increased with an increasing dose of PEREM. At 4 h post feeding, the CH_4_ concentrations in the rumen of the animal were linearly reduced when the dose of pellets was increased (*p* < 0.01). In addition, the inclusion of PEREM did not adversely affect other blood metabolites, namely total protein, creatinine and albumin (*p* > 0.05). Furthermore, microbial crude protein and efficiency of microbial N synthesis were linearly enhanced when increasing levels of PEREM were added. The feeding of PEREM at 150 g/d might be an alternative with the potential to improve rumen fermentation efficiency and reduce the environmental effects produced by ruminants.

## 1. Introduction

Manipulation of the rumen is interesting particularly for increasing rumen efficiency, improving feed utilization and reducing environmental pollution [1]. Strategic feeding of ruminants with pellets containing phytochemical compounds is one approach that could enhance rumen fermentation and decrease greenhouse gases. Furthermore, pelleted feeds enhance the acceptability, density and quality of diets [2]. Previous studies demonstrated that supplementation with pelleting plants rich in saponins and tannins such as raw banana [3], mangosteen (*Garcinia mangostana*) peel [4], Leucaena leaf [5] and banana flower [6] could improve ruminal propionic acid concentration and microbial protein synthesis while reducing the protozoal population and ruminal methane (CH_4_) gas production. Cieslak et al. [7] demonstrated that a reduction in CH_4_ emissions by plants rich in saponins and tannins could be mediated through: (1) a reduction in protozoal populations; (2) the direct interruption of methanogenic bacteria; (3) alterations in the volatile fatty acids (VFA) profile and the ratio of acetate (C2) to propionate (C3); and (4) the interruption of ruminal cellulolytic enzyme activity, thus depressing the nutrient digestibility. However, an experiment was still required to test the suitable sources of saponins and tannins, and the feeding methods required to reduce CH_4_ and stimulate ruminant production [1].

Royal poinciana (*Delonix regia*), a wild plant otherwise called flame of the forest, originated in Madagascar but is now distributed over several countries in the tropical regions, including Thailand. It produces pods containing seeds that are rich in tannins and saponins at 93.1 mg/100 g dry matter (DM) and 12.3 g/kg DM, respectively [8]. A previous study elucidated that royal poinciana seed meal successfully improves rumen fermentation efficiency, reduces protozoa and mitigates CH_4_ concentrations in the rumen of cattle [8]. However, the development of royal poinciana as pellet feed to improve the value added to and to enhance the rumen efficiency of beef cattle needs to be developed.

Thus, the object of this present work was to determine the effects of supplementation with pellets containing royal poinciana seed meal (PEREM) on feed use, ruminal fermentation efficiency, microbial protein synthesis, blood metabolites, and mitigating CH_4_ emission in Thai native beef cattle.

## 2. Materials and Methods

Animals used in the present experiment were approved by the Animal Ethics Committee of Khon Kaen University (record no. ACUC-KKU 34/2559), based on the Ethics of Animal Experimentation of the National Research Council of Thailand.

### 2.1. Pellets Containing Royal Poinciana Seed Meal (PEREM) Preparation and Design

PEREM was prepared from royal poinciana pods that were obtained from the Khon Kaen area. The seeds were collected from pods that were sun-dried for three weeks and then ground to pass through a 0.1 cm sieve (Cyclotech Mill, Tecator, Sweden). Royal poinciana seed meal was mixed with other ingredients (Table 1). The ingredients were put into a pelleting machine and left to sun-dry for 2–3 days to decrease remaining moisture to less than 15% before feeding it to the cattle [9]. 

### 2.2. Treatments, Experimental Design and Animal’s Management

The animals used in this experiment were four male Thai native beef cattle (*Bos indicus*) with initial body weights (BWs) of 125 ± 5.0 kg. Each of the animals were randomly assigned to receive PEREM doses at 0, 50, 100 and 150 g/d, respectively, according to a 4 × 4 Latin square design. Concentrates were fed at 0.5% BW daily, and rice straw was fed *ad libitum*, allowing for 10% of refusals. All diets were fed at 07:00 h and 16:00 h in two equal meals per day. All animals were kept in individual houses, and fresh water and mineral blocks were available at all times. Four 21-day periods were used in this study. All cattle were fed their respective diets in the houses during the first 14 days, whereas during the last 7 days, the animals were transferred to metabolism crates for collection of feces and urine. For the first 2 days, all animals were allowed time to adjust to the crates and feed, and then for the last 5 days, feces and urine were collected to determine the results of the feed use on nitrogen utilization. The chemical compositions of the experimental diets are presented in Table 1.

### 2.3. Sample Collection and Measurements

5% of total fresh fecal samples were collected for daily DM analysis. Another part of the feces was kept in a refrigerator and pooled by cattle at the last day of each period for chemical analysis. The content of DM, crude protein (CP), ash, and acid detergent fiber (ADF) in the samples (feeds, refusal and fecal) was analyzed using the Association of Official Analytical Chemists (AOAC) method [10], whereas neutral detergent fiber (NDF) was determined according to Van Soest et al. [11]. The concentration of condensed tannins and saponins in PEREM was measured by the method of Burns [12] and Wang and Fang [13], respectively. 

On the 21st day of each period, 10 mL samples of blood were taken from the jugular vein at 0 h and 4 h post feeding for detection of blood metabolites. The blood samples were centrifuged at 500× *g* for 10 min at 4 °C and stored at −20 °C until used. Concentrations of blood urea-nitrogen (BUN), albumin and creatinine were measured using a diagnostic kit (L typeWako UN and albumin-HRII, Tokyo, Japan). Plasma creatinine was determined by the Roche Hitachi 912 Plus automatic analyzer (Indianapolis, IN, USA). Total blood protein contents were measured by a refractometer (SPR-Ne; Atago Co., Tokyo, Japan). 

Urinary N in urine was measured by using the AOAC method [10], whereas the concentration of allantoin was detected by high pressure liquid chromatography (HPLC). The concentration of microbial purines absorbed and the efficiency of microbial N synthesis were evaluated from purine derivative excretions, based on the relationship derived by Chen and Gomes [14].

On the same day as blood sampling, approximately 100 mL of rumen fluid was sampled (at 0 h and 4 h post feeding) from the middle part of the rumen by a stomach tube connected to a vacuum pump. The rumen fluid was immediately evaluated for pH and temperature using Hanna Instruments’ HI 8424 microcomputer (Kallang Way, Singapore) after withdrawal. The fluid samples were filtered through 4 layers of cheesecloth. The rumen fluid was divided into 2 portions; in the first portion, 45 mL of rumen fluid was mixed with 5 mL of 1 mol H_2_SO_4_, was centrifuged at 16,000× *g* for 15 min, and then was used for NH_3_-N analysis using the Kjeltech Auto 1030 Analyzer, and the VFA were analyzed using HPLC. The CH_4_ concentration was calculated according to the equation of Moss et al. [15] by using VFA proportions as follows: CH_4_ production = 0.45 (acetate) − 0.275 (propionate) + 0.40 (butyrate).

In the last portion, 1 mL of rumen fluid was fixed with 9 mL solution (10% formalin solution in sterilized 0.9% saline) and used for the determination of total bacteria and the protozoal population using a hemocytometer (Boeco, Hamburg, Germany).

### 2.4. Statistical Measurement

Data were analysed using the MIXED procedure in SAS [16]. The model included the fixed effect of treatment, animal as random effect, and the residual error. Data are presented as least squares means with standard error of the means. When F-tests were significant, single degree of freedom orthogonal contrasts were used to determine linear and quadratic effects of increasing PEREM supplementation. Significance was declared at *p* ≤ 0.05, and tendencies were considered at 0.01 ≤ *p* ≤ 0.05.

## 3. Results and Discussion

### 3.1. Nutritional Contents in PEREM

Table 1 shows the chemical composition of the PEREM product and feed used in the present study. The PEREM contained a high concentration of CP at 25.42%, which could be related to the additional 1% of urea in the ration. A feeding strategy for a diet containing a high dose of urea in the pellets is to provide the cattle with a readily available source of rumen-fermentable starch. Royal poinciana and molasses are ingredients that are high in water-soluble starch and are an inexpensive source of energy in some regions, compared to starchy feeds. This might indicate a more synchronized supply of nitrogen and energy to rumen microorganisms, which would have led to an enhanced microbial protein synthesis in the rumen. Furthermore, the PEREM product consisted of a concentration of 84.75 mg/100 g DM of tannins and 11.04 g/kg DM of saponins, which could be beneficial to rumen manipulation, particularly CH_4_ mitigation in the rumen. The concentration of tannins in the PEREM product was slightly lower than in those previous pellet studies, such as the banana flower power pellet (96 mg/100 g DM; [6]) or mangosteen (*Garcinia mangostana*) peel pellet (99.5 mg/100 g DM; [4]). This might be because the tannins concentration in the raw plants differed among the sources.

### 3.2. Feed Intakes, Nutrient Intake and Digestibility Coefficients

Table 2 shows the intake of feeds, nutrients and digestibility coefficients as affected by the different doses of the PEREM supplement given to the animals. There were no significant differences (*p* > 0.05) on intakes of rice straw, concentrate and total. The total intake ranged from 2.59 to 2.76 kg/d, which was the normal range when the animals were fed on low quality roughage such as rice straw. However, the addition of PEREM may provide more nutrients to supply the rumen microorganisms and support the animal host. With supplementation of PEREM at 50 to 150 g/d, the animals received tannins and saponins for 0.042 to 0.127 mg/100 g DM and 0.006 to 0.017 g/kg DM, respectively. These concentrations of plant metabolites could be beneficial to ruminal fermentation and the reduction of CH_4_. The intake of nutrients did not change among the levels of PEREM supplementation (*p* > 0.05), except for an intake of CP, which was linearly enhanced when increasing the dose of PEREM (*p* < 0.05). Compared to the group that was fed no PEREM, the CP intake increased at 0.04 kg/d when 150 g PEREM was added. This could be because PEREM contains high CP (25.42% DM), resulting in the high CP intake when 150 g was introduced. The high CP intake could provide N available for rumen microbes to synthesize their cells with their energy source. The inclusion of different doses of PEREM did not adversely affect the digestibility of DM, organic matter (OM), CP, NDF and ADF (*p* > 0.05). DM digestibility ranged from 63.05 to 64.69%, which was the normal range in the animals fed the rice straw-based diet. However, Supapong et al. [8] elucidated that DM digestibility was reduced when increasing un-pelleted royal poinciana seed meal doses (*p* < 0.05). This result indicated that plant secondary metabolites in the un-pelleted form might be strongly negatively affecting the rumen microbes and reducing feed digestion, whereas the pelleted form could be more beneficial. Similarly, Norrapoke et al. [4] indicated that nutrient intake and digestibility were unchanged with various doses of mangosteen peel pellet supplementation, while the NDF and ADF digestion tended to decrease. It could possibly be that there is a higher content of tannins and saponins in mangosteen peel pellet than in those with PEREM, thus, inhibiting microbial activity for breaking down fiber [7].

### 3.3. Rumen Ecology and Microorganism Population

Table 3 shows the rumen ecology, ruminal microbes and predominant cellulolytic bacteria in animals fed different levels of PEREM. Adding various doses of PEREM did not alter ruminal pH and ruminal temperature, which were in the suitable range for the microbial enzyme activity of feed digestion. Ruminal pH and temperature ranged from 6.84 to 6.92 and 38.71 °C to 39.20 °C, which was similarly reported by Supapong et al. [8], who investigated under in the same animal conditions. The concentrations of NH_3_-N was significantly different among the various doses of PEREM supplementation (*p* < 0.01). The increasing doses of PEREM supplementation were linearly increased with NH_3_-N concentrations. The supplementation of PEREM at 150 g/d increased the mean NH_3_-N concentration by 6.70 mg/dL when compared to the no-PEREM supplemented group. This may be due to the high CP intake when the PEREM was added, thus leading to a high amount of CP available for microbial breakdown to NH_3_-N in the rumen. Rumen NH_3_ is the main product of protein diet degradation, which appears to have been generally agreed upon, that most of the N utilized by rumen microorganisms comes from the NH_3_ pool in the rumen. Thus, the high levels of CP in the feed can enhance the NH_3_-N content in the rumen. Similarly, Hung et al. [5] indicated that ruminal NH_3_-N concentration was increased with Leucaena leaf pellet supplementation at 450 g/d compared to a no-pellet fed group.

The effect of PEREM supplementation doses on rumen protozoal populations is also shown in Table 3. The increasing doses of PEREM linearly reduced protozoal numbers (*p* < 0.01), with the lowest concentration when PEREM was added at 150 g. Compared to the no-PEREM fed group, supplementation of PEREM at 150 g reduced the population of protozoa by 76.64% when the rumen fluid was sampled at 4 h after feeding. The reduction of the protozoal population could be due to the tannins and saponins contained in the PEREM may inhibit the activity of the rumen protozoa, likely by binding the proteins and enzymes of the protozoal membranes as noted by Cieślak et al. [7]. This agrees with the findings of Kang et al. [6], who revealed that the supplementation of banana flower powder pellets at 60 g/kg of substrate in an in vitro study showed reduced protozoal numbers by 58.01% compared to the feed with no pellets added. In addition, the dairy cows receiving 300 g/d of mangosteen peel pellet had decreased protozoal counts by 27.62%, while Hung et al. [5] found protozoa to be reduced by 36.11% when cattle received Leucaena leaf pellets at 450 g/d. Moreover, the population of total bacterial counts were not adversely affected by PEREM supplementation (*p* > 0.05). Thus, supplementing with pellets containing plant secondary compounds could be beneficial in the reduction of protozoal concentrations and the maintenance of the bacterial population in the rumen.

### 3.4. Ruminal VFA and CH_4_ Estimation

The calculation of the concentration of ruminal VFA and CH_4_ calculation in animals fed with different doses levels of PEREM is shown in Table 4. PEREM supplementation did not change (*p* > 0.05) the concentrations of acetic acid (C2) or butyric acid (C4) or the ratio of C2 to propionic acid (C3). Nevertheless, the total VFA and C3 content were changed among PEREM levels (*p* < 0.05), which were linearly enhanced with an increasing dose of PEREM. At 4 h post feeding, they were increased by 10.43 mmol/L and 2.2 mol/100 mol of total VFA and C3, respectively, when compared to the 0 g PEREM supplementation. These increases could be related to the fact that PEREM contains soluble energy sources such as starch and molasses that may supply C3 for syntheses by the rumen microorganisms. In addition, Anantasook et al. [17] indicated that the expected shift of hydrogen from the CH_4_ pathway could provide C3 production. Thus, an alternative electron sink for a metabolic route to dispose of the reduced power has to happen. Furthermore, Newbold et al. [18] proposed that the succinate-C3 pathway could possibly lead to C3 production. 

At 4 h post feeding, the CH_4_ concentration in the rumen of the animals significantly differed among those given \ various doses of PEREM (*p* < 0.01), which were linearly reduced when the dose of pellets was increased. Compared to the nonsupplemented group, the group fed PEREM at 100 g showed reduced CH_4_ concentrations by about 16.81%, whereas the highest CH_4_ reduction of about 25.03% was found when 150 g was added. The reduction percentage of CH_4_ was close to the previous study by Supapong et al. [8], who supplemented 270 g of DM un-pelleted royal poinciana seed and decreased CH_4_ by about 19.12% when compared to the nonsupplemented group. In addition, feeding on pelleting plants rich in saponins and tannins such as raw banana, mangosteen peel, Leucaena leaf or banana flower also reduced the CH_4_ concentration in in vitro and in vivo studies [3,4,5,6]. This might be because the pellets containing plants rich in saponins and tannins reduced protozoal populations and methanogenic bacteria [7]. In addition, changes in the VFA profile and the ratio of the C2 to C3 could be another factor affecting the CH_4_ reduction [19,20,21].

### 3.5. Blood Metabolites

Blood metabolites in the animals fed different levels of PEREM are presented in Table 5. BUN concentration was significantly different between animals fed with different levels of PEREM supplementation (*p* < 0.05). Increasing doses of PEREM were linearly increased with BUN, which was highest when 150 g of PEREM was added. BUN is an indicator for determining protein utilization efficiency and is related to protein intake and ruminal NH_3_-N concentrations. In the present experiment, the increasing concentration of ruminal NH_3_-N for 150 g PEREM led to an increased concentration of BUN. However, the concentration of BUN was in the normal range (11.54 to 16.89 mg/dL), which also was reported by Supapong at al. [8]. In addition, the inclusion of PEREM did not adversely affect other blood metabolites, namely total protein, creatinine and albumin, indicating that PEREM could be supplemented to beef cattle as a rumen modifier.

### 3.6. Nitrogen Balances and Microbial Protein Synthesis

Table 6 shows nitrogen utilization, excretion of urinary derivatives, microbial crude protein (MCP) and efficiency of microbial N synthesis (EMNS) in the Thai native beef cattle fed different doses of PEREM. There were significant differences among the levels of PEREM on all parameters of N balances (*p* < 0.05), except urinary N (*p* > 0.05), which was unchanged. N intake, N absorption and N retention were linearly enhanced with increasing levels of PEREM and were highest when PEREM was supplemented at 150 g. Allantoin excretion and absorption were changed by the levels of PEREM supplementation. Furthermore, MCP and EMNS were linearly enhanced when increasing levels of PEREM were added. Supplementation of PEREM at 150 g produced higher increases than other levels of supplementation. This might be because the tannins and saponins in PEREM have the potential to improve the ruminal fermentation toward maximizing microbial protein synthesis [17]. Furthermore, tannins could support a synchronized a release of nitrogen and carbohydrates, which is responsible for microbial efficiency enhancement.

## 4. Conclusions

Supplementation of PEREM at 150 g/d in Thai native beef cattle could enhance CP intake, total VFA, propionic acid, nitrogen balance, MPC and EMNS, while reducing the protozoal number and methane concentration. Thus, feeding with PEREM might be an alternative with the potential to improve rumen fermentation efficiency and reduce the environmental effects produced by ruminants. However, further research on the diversity of methanogenic bacteria as affected by PEREM is required.

## Figures and Tables

**Table 1:** Ingredient and chemical composition of concentrate, pellets containing royal poinciana seed meal (PEREM) and rice straw.

Items	Concentrate	PEREM	Rice Straw
Ingredients, %DM			
Cassava chips	55.50	-	
Rice bran	11.00	-	
Coconut meal	13.00	-	
Palm kernel meal	13.40	-	
Royal poinciana seeds meal *	-	90.00	
Cassava starch	-	5.00	
Minerals and vitamins ^†^	0.50	1.00	
Sulfur	0.50	1.00	
Urea	2.60	1.00	
Salt	1.00	1.00	
Molasses	2.00	1.00	
Chemical composition			
Dry matter, %	93.53	96.73	94.54
Organic matter, %DM	92.75	93.61	93.12
Crude protein, %DM	14.04	25.42	2.83
Neutral detergent fiber, %DM	11.85	18.95	66.87
Acid detergent fiber, %DM	7.97	12.31	43.37
Tannins, mg/100 g DM	-	84.75	-
Saponins, g/kg DM	-	11.04	-

* Royal poinciana seeds meal contains 89.5% dry matter (DM), 21.2% crude protein (CP) 34.2% neutral detergent fiber (NDF), 24.5% acid detergent fiber (ADF), 93.1 mg/100 g DM of tannins and 12.3 g/kg DM of saponins. ^†^ Minerals and vitamins (each kg contains): Vitamin A: 10,000,000 IU; Vitamin E: 70,000 IU; Vitamin D: 1,600,000 IU; Fe: 50 g; Zn: 40 g; Mn: 40 g; Co: 0.1 g; Cu: 10 g; Se: 0.1 g; I: 0.5 g.

**Table 2:** Influence of different levels of pellets containing royal poinciana seed meal (PEREM) on feed intake, nutrient intake and apparent digestibility in Thai native beef cattle.

Item	Supplementation of PEREM, g DM				SEM	Contrast	
	0	50	100	150		Linear	Quadratic
DM intake							
Rice straw							
kg/day	1.93	1.96	2.00	1.95	0.16	0.86	0.82
g/kg BW^0.75^	47.41	47.88	48.93	48.17	2.70	0.79	0.83
Concentrate							
kg/day	0.66	0.66	0.66	0.65	0.02	0.86	0.92
g/kg BW^0.75^	16.08	16.10	16.09	16.06	0.16	0.92	0.88
PEREM							
kg/day	0.00	0.05	0.10	0.15	-	-	-
g/kg BW^0.75^	0.00	1.16	2.32	3.51	-	-	-
Total intake							
kg/day	2.59	2.67	2.76	2.75	0.18	0.50	0.83
g/kg BW^0.75^	63.23	65.14	67.34	67.73	2.83	0.26	0.80
Nutrient intake, kg/d							
Dry matter	2.59	2.67	2.76	2.75	0.18	0.50	0.83
Organic matter	2.50	2.58	2.67	2.67	0.17	0.48	0.83
Crude protein	0.16 ^a^	0.17 ^a^	0.19 ^a^	0.20 ^b^	0.01	0.02	0.88
aNeutral detergent fiber	1.65	1.69	1.74	1.72	0.13	0.70	0.83
Acid detergent fiber	1.04	1.07	1.10	1.09	0.08	0.64	0.81
Digestibility coefficients, %							
Dry matter	64.69	64.05	63.48	63.05	2.88	0.22	0.72
Organic matter	68.16	67.22	67.11	66.95	2.57	0.39	0.97
Crude protein	62.24	62.18	62.08	62.62	2.33	0.92	0.90
aNeutral detergent fiber	55.27	55.04	54.64	53.24	4.02	0.73	0.89
Acid detergent fiber	46.11	45.50	45.08	44.38	4.70	0.66	0.99

^a,b^ Values within the same row not bearing a common superscript differ (*p* < 0.05).

**Table 3:** Rumen ecology, microorganism and predominant cellulolytic bacteria in Thai native beef cattle fed different levels of pellets containing royal poinciana seed meal (PEREM).

Item	Supplementation of PEREM, g DM				SEM	Contrast	
	0	50	100	150		Linear	Quadratic
Rumen ecology							
Ruminal pH							
0 h post feeding	6.93	6.90	6.93	6.95	0.10	0.80	0.69
4 h post feeding	6.73	6.90	6.90	6.86	0.05	0.17	0.12
Mean	6.84	6.90	6.92	6.90	0.06	0.42	0.56
Ruminal temperature, °C							
0 h post feeding	38.53	38.01	38.52	38.61	0.24	0.42	0.27
4 h post feeding	39.54	39.32	39.43	39.82	0.19	0.30	0.16
Mean	39.01	38.71	38.91	39.20	0.18	0.29	0.15
NH_3_-N concentration, mg/dL							
0 h post feeding	12.71 ^a^	14.01 ^b^	15.70 ^c^	19.50 ^d^	0.16	<0.01	<0.05
4 h post feeding	16.82 ^a^	18.51 ^b^	20.63 ^c^	23.52 ^d^	0.14	<0.01	0.05
Mean	14.80 ^a^	16.33 ^b^	18.21 ^c^	21.50 ^d^	0.11	<0.01	<0.05
Ruminal microbes, cell/mL							
Protozoa × 10^6^							
0 h post feeding	10.51 ^a^	5.31 ^b^	4.43 ^bc^	2.51 ^c^	0.56	<0.01	<0.05
4 h post feeding	12.50 ^a^	6.82 ^b^	5.43 ^bc^	2.92 ^c^	0.91	<0.05	0.13
Mean	11.51 ^a^	6.01 ^b^	4.93 ^b^	2.73 ^c^	0.6	<0.01	<0.05
Bacteria × 10^8^							
0 h post feeding	13.50	12.88	13.65	12.99	1.05	0.88	0.86
4 h post feeding	19.88	18.96	20.15	19.65	1.88	0.12	0.85
Mean	16.69	15.92	16.90	16.32	1.51	0.35	0.83

^a–d^ Values within the same row not bearing a common superscript differ (*p* < 0.05).

**Table 4:** Concentrations of ruminal volatile fatty acid (VFA) profiles and methane (CH_4_) estimation in Thai native beef cattle fed with various levels of pellets containing royal poinciana seed meal (PEREM).

Item	Supplementation of PEREM, g DM				SEM	Contrast	
	0	50	100	150		Linear	Quadratic
Total VFA, mmol/L							
0 h post feeding	102.00	103.65	106.68	106.85	3.06	0.21	0.15
4 h post feeding	106.04 ^a^	110.29 ^b^	116.38 ^c^	116.47 ^c^	0.46	<0.01	<0.01
Mean	104.02 ^a^	106.97 ^b^	111.53 ^c^	111.66 ^c^	0.23	<0.01	<0.01
VFA profiles, mol/100 mol							
Acetic acid							
0 h post feeding	65.31	64.06	63.95	62.24	2.22	0.11	0.35
4 h post feeding	67.70	66.37	67.16	66.95	3.37	0.41	0.18
Mean	66.50	65.22	65.55	64.59	2.14	0.21	0.27
Propionic acid							
0 h post feeding	22.2 ^a^	23.19 ^b^	24.01 ^c^	25.07 ^d^	0.10	<0.01	0.75
4 h post feeding	23.11 ^a^	24.09 ^b^	25.08 ^c^	25.31 ^c^	0.18	<0.01	0.08
Mean	22.65 ^a^	23.64 ^b^	24.54 ^c^	25.19 ^d^	0.11	<0.01	0.18
Butyric acid							
0 h post feeding	12.49	12.69	12.05	12.70	1.50	0.99	0.48
4 h post feeding	9.19	9.54	7.77	7.74	1.20	0.21	0.39
Mean	10.84	11.11	9.91	10.22	1.13	0.81	0.89
Acetic/propionic acid ratio	2.94	2.76	2.67	2.57	0.52	0.51	0.06
CH_4_ estimation, mM/L							
0 h post feeding	28.79	27.05	26.43	26.26	0.57	0.11	0.73
4 h post feeding	36.23 ^a^	33.03 ^b^	30.14 ^c^	27.16 ^d^	0.94	<0.01	0.08
Mean	32.51 ^a^	30.04 ^b^	28.28 ^c^	26.71 ^d^	0.68	<0.01	0.16

^a–d^ Values within the same row not bearing a common superscript differ (*p* < 0.05).

**Table 5:** Blood metabolites in Thai native beef cattle fed different levels of pellets containing royal poinciana seed meal (PEREM).

Item	Supplementation of PEREM, g DM				SEM	Contrast	
	0	50	100	150		Linear	Quadratic
Blood metabolites							
Blood urea-N concentration, mg/dL							
0 h post feeding	10.25	10.25	11.12	11.35	0.50	0.53	0.41
4 h post feeding	11.54 ^a^	13.55 ^b^	14.87 ^c^	16.89 ^d^	0.35	0.01	0.09
Mean	10.90 ^a^	11.90 ^a^	13.00 ^b^	14.12 ^b^	0.24	0.04	0.51
Total protein, g/dL							
0 h post feeding	1.46	1.42	1.33	1.36	0.08	0.34	0.70
4 h post feeding	1.40	1.42	1.46	1.48	0.12	0.61	0.98
Mean	1.43	1.42	1.40	1.42	0.09	0.92	0.83
Creatinine, mg/dL							
0 h post feeding	5.98	5.78	6.10	6.33	0.17	0.12	0.26
4 h post feeding	6.13	6.10	6.28	6.38	0.14	0.19	0.67
Mean	6.05	5.94	6.19	6.35	0.13	0.10	0.34
Albumin, g/dL							
0 h post feeding	3.03	2.95	3.13	2.90	0.08	0.57	0.36
4 h post feeding	2.95	3.20	3.00	3.05	0.14	0.87	0.49
Mean	2.99	3.08	3.06	2.98	0.05	0.84	0.15

^a–d^ Values within the same row not bearing a common superscript differ (*p* < 0.05).

**Table 6:** Effects of different levels of pellets containing royal poinciana seed meal (PEREM) on N utilization, excretion of urinary derivatives (PD), microbial crude protein and efficiency of microbial N synthesis of Thai native beef cattle.

Item	Supplementation of PEREM, g DM				SEM	Contrast	
	0	50	100	150		Linear	Quadratic
Nitrogen (N) balance, g/d							
N intake (NI)	23.65 ^a^	25.62 ^b^	27.54 ^c^	29.02 ^d^	0.14	<0.01	0.14
N fecal	9.55 ^ac^	10.35 ^ab^	10.92 ^b^	9.05 ^c^	0.34	0.55	<0.01
N urine	6.10	5.96	5.10	4.88	0.46	0.07	0.94
N absorption	14.10 ^a^	15.27 ^b^	16.62 ^c^	19.97 ^d^	0.23	<0.01	<0.01
N retained (NR)	8.00 ^a^	9.31 ^a^	11.52 ^b^	15.10 ^c^	0.53	<0.01	0.08
PD, mmol/d							
Allantoin excretion	121.02 ^a^	125.65 ^a^	131.2 ^ab^	135.44 ^b^	3.98	0.05	0.78
Allantoin absorption	115.89 ^a^	119.35 ^a^	125.77 ^b^	129.89 ^ab^	2.65	0.03	0.25
Microbial crude protein *, g/d	413.34 ^a^	429.15 ^a^	448.11 ^ab^	462.59 ^b^	10.8	0.02	0.54
EMNS ^†^, g N/kg OMDR	37.32 ^a^	38.07 ^a^	39.06 ^ab^	41.04 ^b^	0.68	0.04	0.09

* Microbial crude protein (MCP) (g/d) =3.99 × 0.856 × mmoles of purine derivatives excreted [22]. ^†^ Efficiency of microbial N synthesis (EMNS, g/kg of organic matter (OM) digested in the rumen (OMDR) = ((MCP (g/d) × 1000)/DOMR (g)), assuming that rumen digestion was 650 g/kg OM of digestion in total tract. ^a–d^ Values within the same row not bearing a common superscript differ (*p* < 0.05).

## References

[1] Wanapat M., Kang S., Polyorach S. (2013). Development of feeding systems and strategies of supplementation to enhance rumen fermentation and ruminant production in the tropics. J. Anim. Sci. Biotechnol..

[2] Südekum K.H., Schröder A., Fiebelkorn S., Schwer R., Thalmann A. (2008). Quality characteristics of pelleted compound feeds under varying storage conditions as influenced by purity and concentration of glycerol from biodiesel production. J. Anim. Feed Sci..

[3] Lunsin R., Wanapat M., Wachirapakorn C., Navanukraw C. (2010). Effects of pelleted cassava chip and raw banana (cass-bann) on rumen fermentation and utilization in lactating dairy cows. J. Anim. Vet. Adv..

[4] Norrapoke T., Wanapat M., Wanapat S. (2012). Effects of protein level and mangosteen peel pellets (mago-pel) in concentrate diets on rumen fermentation and milk production in lactating dairy crossbreds. Asian-Australas. J. Anim. Sci..

[5] Hung L.V., Wanapat M., Cherdthong A. (2013). Effects of Leucaena leaf pellet on bacterial diversity and microbial protein synthesis in swamp buffalo fed on rice straw. Livest. Sci..

[6] Kang S., Wanapat M., Bounnaxay V. (2016). Supplementation of banana flower powder pellet and plant oil sources on in vitro ruminal fermentation, digestibility, and methane production. Trop. Anim. Health Prod..

[7] Cieślak A., Zmora P., Matkowski A., Nawrot-Hadzik I., Pers-Kamczyc E., El-Sherbiny M., Bryszak M., Szumacher-Strabe M. (2016). Tannins from *Sanguisorba officinalis* affect in vitro rumen methane production and fermentation. J. Anim. Plant Sci..

[8] Supapong C., Cherdthong A., Seankamsorn A., Khonkhaeng B., Wanapat M., Uriyapongson S., Gunun N., Gunun P., Chanjula P., Polyorach S. (2017). In vitro fermentation, digestibility and methane production as influenced by *Delonix regia* seed meal containing tannins and saponins. J. Anim. Feed Sci..

[9] Seankamsorn A., Cherdthong A., Wanapat M., Supapong C., Khonkhaeng B., Uriyapongson S., Gunun N., Gunun P., Chanjula P. (2017). Effect of dried rumen digesta pellet levels on feed use, rumen ecology, and blood metabolite in swamp buffalo. Trop. Anim. Health Prod..

[10] Association of Official Analytical Chemists (AOAC) (1998). Official Analytical Chemists.

[11] Van Soest P.J., Robertson J.B., Lewis B.A. (1991). Methods for dietary fiber neutral detergent fiber, and non-starch polysaccharides in relation to animal nutrition. J. Dairy Sci..

[12] Burns R.E. (1971). Method for estimation of tannin in the grain sorghum. Agron. J..

[13] Wang Z., Fang Y.Z. (2004). Analysis of sugars in traditional Chinese drugs. J. Chromatogr. B.

[14] Chen X.B., Gomes M.J. (1995). Estimation of Microbial Protein Supply to Sheep and Cattle Based on Urinary Excretion of Purine Derivative-An Overview of the Technique Details.

[15] Moss A.R., Jouany J.P., Newbold J. (2000). Methane production by ruminants: its contribution to global warming. Ann. Zootech..

[16] SAS (1998). User’s Guide: Statistics, Version 6.

[17] Anantasook N., Wanapat M., Gunun P., Cherdthong A. (2016). Reducing methane production by supplementation of Terminalia chebula RETZ. containing tannins and saponins. Anim. Sci. J..

[18] Newbold C.J., Lopez S., Nelson N., Ouda J.O., Wallace R.J., Moss A.R. (2005). Propionate precursors and other metabolic intermediates as possible alternative electron acceptors to methanogenesis in ruminal fermentation *in vitro*. Br. J. Nutr..

[19] Patra A.K., Kamra D.N., Agarwal N. (2010). Effects of extracts of spices on rumen methanogenesis, enzyme activities and fermentation of feeds *in vitro*. J. Sci. Food Agric..

[20] Liu J.X., Susenbeth A., Südekum K.H. (2002). In vitro gas production measurements to evaluate interactions between untreated and chemically treated rice straws, grass hay, and mulberry leaves. J. Anim. Sci..

[21] Cherdthong A., Khonkhaeng B., Foiklang S., Wanapat M., Gunun N., Gunun P., Chanjula P., Polyorach S. (2019). Effects of supplementation of *Piper sarmentosum* leaf powder on feed efficiency, rumen ecology and rumen protozoal concentration in Thai native beef cattle. Animals.

[22] Cherdthong A., Wanapat M., Wachirapakorn C. (2011). Influence of urea-calcium mixtures as rumen slow-release feed on in vitro fermentation using gas production technique. Ach. Anim. Nutr..

